# Experimental Evaluation of Suitability of Selected Multi-Criteria Decision-Making Methods for Large-Scale Agent-Based Simulations

**DOI:** 10.1371/journal.pone.0165171

**Published:** 2016-11-02

**Authors:** Petr Tučník, Vladimír Bureš

**Affiliations:** Faculty of Informatics and Management, University of Hradec Králové, Rokitanského 62, Hradec Králové, Czech Republic; Universite Toulouse 1 Capitole, FRANCE

## Abstract

Multi-criteria decision-making (MCDM) can be formally implemented by various methods. This study compares suitability of four selected MCDM methods, namely WPM, TOPSIS, VIKOR, and PROMETHEE, for future applications in agent-based computational economic (ACE) models of larger scale (i.e., over 10 000 agents in one geographical region). These four MCDM methods were selected according to their appropriateness for computational processing in ACE applications. Tests of the selected methods were conducted on four hardware configurations. For each method, 100 tests were performed, which represented one testing iteration. With four testing iterations conducted on each hardware setting and separated testing of all configurations with the–server parameter de/activated, altogether, 12800 data points were collected and consequently analyzed. An illustrational decision-making scenario was used which allows the mutual comparison of all of the selected decision making methods. Our test results suggest that although all methods are convenient and can be used in practice, the VIKOR method accomplished the tests with the best results and thus can be recommended as the most suitable for simulations of large-scale agent-based models.

## Introduction

In the realm of socio-economic systems, problem solving is mostly related to the necessity for individual agents to make right decisions. In most cases, the operational level of decision-making can be handled by standard optimization algorithms, as these problems mostly require only the optimized solution, and decision-making is generally specialized and not overwhelmingly complex. Nevertheless, decision-making on both tactical and strategic levels is generally dependent on a large number of factors and is characterized by the pursuit of parallel goals, and the decision-making component is expected to adapt to changing conditions because economic environments are typically dynamic and complex in nature. Therefore, these environments are modelled with the help of various techniques or approaches. The traditional analytical approach to modelling of economic systems has several weak points [1]. Therefore, modelling based on systems dynamics principles [2] or agent-based techniques has recently emerged as a viable alternative. Tied to the latter, a new discipline termed Agent-based Computational Economics (ACE) has been established. In this field of study, multi-agent economic systems comprise multiple intelligent agents that interact to solve problems that might be beyond the capabilities of a single agent or system [3]. Hence, individual agents represent economic entities of various types, and the performance of the whole system is the result of a large number of their mutual interactions. ACE models are constructed through a bottom-up approach, i.e., there is no centralized control or decision-making. However, this approach causes one of the main issues of the agent-based approach, especially when large-scale models are developed and used for simulations. As the square law of computation indicates [4], as the number of agents increases, the computation complexity rises disproportionately. This limitation forces users to apply as effective methods or techniques as possible. In case of decision-making agents it encourages the exploration of the possibility of applying multi-criteria decision-making as the main control mechanism on both the individual and collective level of multi-agent systems. Unfortunately, there is a very limited number of studies published that would explicitly deal with application of multi-criteria decision-making (MCDM) methods in multi-agent models. However, there are studies connecting decision-making at the general level and agent-based modelling. For instance, while Yu and Xu [5] deal with graph-based multi-agent decision making, Nguyen et al. [6] investigate decision-making agents in rice pest risk management. Obviously, exploration of suitability of particular MCDM methods for large-scale agent-based simulations represents a significant research gap and associated experiments can offer results with a high level of value added and novelty.

This study follows the seminal work of Triantaphyllou [7], who states that the fundamental research question is "which is the best method for a given problem?” Similarly, Peng et al. [8] state that “it is a challenging task to decide, which MCDM method(s) are suitable for a problem”. In case of this study, the problem is a decision-making process performed by agents in large-scale agent-based systems, which represents the main research construct of this study. Thus, rather than development of a new MCDM method suitable for decision-making in multi-agent systems or adjustment of the existing one, it is more suitable to focus on several existing formal methods that might be applied in this specific decision-making environment. This study is based on two main assumptions, which existence and thus relevancy was verified by review of research papers published in three scientific databases–Scopus, ScienceDirect and Web of Knowledge. First, no study comparing the computational effectiveness of selected multi-criteria decision-making methods in large-scale agent-based systems has been conducted yet. Second, agent-based models are mostly developed and simulated on standard personal computers, laptops, or notebooks. Although there is an intensive research focused on usage of distributed environments, high performance (HPC) and high throughput (HTC) compute platforms, Grid or clouds ([9] or [10]), we consider it as specific and mostly associated with particular research conducted in the information technology realm. It can be assumed that agent-based models are mostly built and simulated on commodity compute resources and large-scale models are mostly developed and simulated with the help of similar configurations as well. Although the aforementioned review of studies revealed that absolute majority of models are presented without description of hardware configurations that was used for modelling and simulations, there are two main prevailing reasons for this assumption. First, Anylogic as one of the leading agent-based platform provider states system requirements at its web pages. Even when AnyLogic represents quite sophisticated set of tools that focus on various modelling paradigms, requirements are in concordance with equipment of standard low-cost computers or laptops (dated to 2015/2016). Second, existing literature supports this assumption as well. For instance, the international group of researchers states that “recent advances in computing hardware such as the wide availability of multi-core CPUs and increased main memory capacities make it possible to investigate population-scale phenomena using commodity compute resources.” [11]. Based on these two assumptions the main research question is formulated as follows: Which from the selected MCDM methods is the most suitable (least time demanding) for application in large-scale agent-based simulations using commodity compute resources? The main objective of this manuscript is to compare computational effectiveness of the WPM, TOPSIS, VIKOR and PROMETHEE methods during the simulation of a specific decision making situation in economic the large-scale agent-based system. In this context, the suitability constitutes time demands associated with runtimes required for execution of the method. The main working hypothesis states that among these four methods, one surpasses the others in terms of suitability as defined above. The subsequent parts of the paper are structured as follows. After the introductory section, the materials and methods are presented. More specifically, the agent-based computational economy used as a platform for the experiments is introduced and the specific experimental software and hardware settings are described. Further, description of the theoretical basis of the decision-making methods applied in this study is provided. The third section presents the results and discusses them in a broader context. Consequently, study limitations and further research paths are outlined. The final section presents the conclusions.

## Materials and Methods

At the beginning of the research, several platforms for development of multi-agent systems were available for the implementation of the model. In order to find the most suitable platform, following criteria were defined, according to basic requirements:

- Geographical Information Systems (GIS) data support, Win OS compatibility,
- orientation on economic models (preferably),
- capacity to handle large-scale models, i.e. models with several thousands of agents or more,
- deliberative (or variable) decision-making support,
- object-oriented structure, preferably Java-based,
- various simulation approaches,
- built-in optimization support for experiments.

Based upon these requirements, several platforms were taken into consideration.

1. NetLogo (https://ccl.northwestern.edu/netlogo/)–the NetLogo platform is a tool for development of agent-based models, available free of charge at the Northwestern Center for Connected Learning and Computer Based Modeling. NetLogo uses simplified representation of the environment (patches organized into a grid) and agents (turtles) and while it is in general possible to use GIS data and employ large number of agents in a model at the same time, it does not offer sufficient complexity for decision making capabilities of agents for the intended purposes and extent of the model. Although implementation is relatively easy, NetLogo can be perceived as slightly obsolete today. While unsuitable for our needs, many successful models were implemented in NetLogo, and developer provides extensive library of implemented models for the user`s convenience.
2. JADE (http://jade.tilab.com/)–JADE (Java Agent Development Framework) is an open source platform fully implemented in Java language. It also fully complies with the FIPA specifications and offers distribution of models over several machines, if needed. However, for the large-scale models, a communication bottleneck exists since all requests are handled by single facilitator entity at certain point (which is an integral part of the Jade communication protocol). Although environment works efficiently with smaller number of agents, for higher number of agents (100k and more) there are severe limitations in model performance. JADE also lacks native support for the GIS data processing.
3. EKI One/MASA platforms (http://masa-group.biz/)–the commercial EKI One platform—originally developed by Artificial Technology GmbH—had integrated Unity support, a Sandbox module for prototyping, and used Lua programming language for scripting. The first version of the model has been partially implemented in this environment, but since the developer company was bought by a competitor (MASA), this platform`s support and further development was terminated. A triplet of platforms was later introduced by the MASA group, namely SWORD (specialized on military logistics and simulations), LIFE (character design and behavior), and SYNERGY (development of DSS for emergency management). Being out of scope for our research, this branch of platforms is no longer used for implementation.
4. AnyLogic (http://www.anylogic.com/)–AnyLogic is a general purpose agent-oriented commercial platform which supports three of the most common modeling paradigms today: (i) agent-based modeling, (ii) discrete event, and (iii) system dynamics simulations. Similar to NetLogo in provision of library of examples and excellent technical support for users, AnyLogic allows implementation of models of various complexity and scale. Supply chain and logistics, manufacturing and production, transportation and warehousing, social processes, or strategic planning and management represent intended application areas for this platform. Moreover, variety of basic functionalities is already well-prepared in AnyLogic libraries, such as pathfinding, GIS support, distribution functions, etc. which makes development of models faster and easier. Altogether, AnyLogic is found to be the most suitable, modern platform available, well-fitting to the needs determined by this study requirements.

The list of agent-based modeling platforms is obviously not complete, for there is a plethora of platforms and toolkits (ADK, Brahms, GAMA, MASON etc.) available on the market. The selection of methods applied in this manuscript covers only platforms closely related to this particular research. The list actually represents only a tip of an iceberg, since multi-agent modeling has become very popular recently and there are literally dozens of platforms available. More thorough survey of agent-based modeling software in general is provided by Nicolai & Madey [12]. Less general and more domain-oriented is recent paper of Hmida that focuses on implementing various MAS architectures in supply chains [13]. Interesting perspective also offers Wang`s paper [14], which deals with smart factories and a strategic initiative called “Industrie 4.0” (German equivalent to Industry 4.0 in English), which was proposed and adopted by the German government as part of the “High-Tech Strategy 2020 Action Plan” [15]. Other studies utilizing agent approach focus mainly on various sub-topics which may be also considered relevant, e.g. more technical view with emphasis on application of engineering approaching in developing simulation models [16], management of SME`s supply chains via MAS outsourcing [17], or MAS supply networks in Industry 4.0 [18].

### Simulation platform

The experiment addresses the computational effectiveness of single multi-criteria decision-making methods without strict relation to other aspects of the economic model. As the model’s functionality, usability, validity, and robustness have already been proved, only a brief description is provided in this section. Additional details associated with the platform can be found in [1].

The economic system has been developed for several months in the AnyLogic software package, v. 7.1.2 x64, with the intention to create a model of real economic systems for use as a suitable platform for various experiments. The main purpose of the model is to represent/emulate an economy at the macro-level, with attention focused especially on the formation of supply chains, consumption, and market interactions. The model implements several types of agents representing:

- consumers (C-agents who consume goods and services and generate a workforce),
- production units (M-agents who harvest and mine the basic production inputs, and industrial F-agents who transform inputs and semi-products to semi-products and products),
- services providers (S-agents offer various services),
- energy providers (E-agents who generate energy for all other agents),
- transportation units (T-agents who ensure transport services).

Furthermore, there is a meta-agent available in a form of colony (COL, who provides public services on macro-level, such as social and health care services, or security, collect taxes and realize public policies). The model requirements and its particularization progressively lead to the implementation of decision-making performed at the individual, group and collective levels. To respect basic prerequisites of ACE model development [19], the government level (COL) does not play the role of a centralized control entity but creates general constrictions or support according to selected policies or priorities (e.g., tax levels, tax relief, support of green technologies, subsidies for selected investments).

The most relevant features of the model are as follows:

- The model consists of a large number of agents (one geographical region is typically represented by more than 10.000 agents).
- The model has complete numerical and symbolic representation.
- From the macro-economic perspective, the variety of possible actions of given economic entities in the model can be perceived as limited.
- Optimization problems at the operational level are handled by standard techniques and algorithms associated with operations management.

Although there are three levels of decision-making in the system, and each level is specific with regard to the input data used and the variety of solutions considered, the mechanics of decision-making remains basically the same, and thus the same decision-making principle is intended for application at all three levels (with respective variations of input data, decision criteria, variants etc.). The main design principle of the model is grounded in the repeated use of standardized modules of identical (or very similar) construction, which makes consequent testing, verification, and overall implementation significantly easier.

### Experimental set-up

The experimental testing of the selected methods is associated with the decision-making of companies (Factory-agents/Mining-agents/Store-agents/Energy-agents/Transport-agents) who need to select the most appropriate candidates for a working position, i.e. decide upon set of agents (Consumer-agents) available in the economic model, who are offered through the labor market, and select the best one according to specialization and work-experience requirements specific for the respective job. Similar approach is used in the model e.g. for establishment of the optimal consumption of goods and services (i.e. consumer basket related decisions), establishing business relationships between agents, such as finding the best supplier, and other decision making situations of economic nature.

The decision-making scenario used for experiments was adopted from Ishizaka & Nemery [20], and generalized in description. Values taken from the original recruitment case study describe selection among four candidates with criteria: personality (weight = 0.1), living abroad (weight 0.4), written test (weight = 0.3), and work experience (weight = 0.2). The case study values were given for criteria personality/living abroad/written test/work experience (respectively): Person #1: 7/9/9/8; Person #2: 8/7/8/7; Person #3: 9/6/7/12; Person #4: 6/11/9/12. Only indifference (Q) and preference (P) thresholds (see the description of the Promethee method below) were determined by the authors. Values of Q and P for criteria Communication skills (score 1–10), Years of living abroad (years), Written test (score 1–10), and Work experience (years) were set to 1;2, 2,4; 1,2; and 2,3 respectively.

In practice, the process is implemented with the help of the abstract class ***DecisionMethod***. This class contains attributes of a decision-making table (alternatives, criteria, criteria weights and values). However, information about maximization or minimization is not included in this class because not all methods need it for their calculations. Furthermore, elements related to the results of multi-criteria decision-making are included. Whereas ***result*** represents the best compromise alternative, ***listOfResults*** provides the complete list of alternatives based on the results obtained. Next, the class comprises methods for the access and setting of particular attributes. Naturally, this class represents the parent class of further classes associated with particular decision-making methods. These specific classes also implement the interface ***IDecision*** for interaction of the user with the simulation environment.

More specifically, all specific classes associated with particular decision-making methods implement given algorithms (see section Calculations below). In the case of the ***Wpm*** class, the constructor requires alternatives, criteria, criteria weights and information about minimization or maximization. Moreover, parameters in the form of a list are required due to the possibility of relatively high numbers of both alternatives and criteria. In addition to the ***Wpm*** class, the ***Topsis*** class addresses normalization. Ideal normalization is selected for the experiment. The selection of the best and the worst evaluations of single criteria form the normalized weighted matrix, which is used for the determination of ideal and negative solutions. The constructor of the ***Vikor*** class requires identical parameters to the ***Topsis*** class. In addition to all previous classes, the ***Promethee*** class constructor requires preference and indifference values in the form of a two-dimensional array. While the array’s first column comprises indifference borders of particular criteria, the second column contains preference borders.

The user interface and available settings modes were created. Furthermore, a menu for the determination of a specific decision-making method and the number of conducted experimental runs was developed.

As already stated in the introductory section of this paper, description of hardware settings applied in published studies is very often missing. Therefore, the experimental testing was performed on four hardware settings (HWs) in order to outline influence of hardware on simulation results. The virtual environment was the only application running on the HWs during the experiment. HWs details are presented in Table 1.

**Table 1:** Hardware settings.

	CPU	RAM	OS	Anylogic	Heap size
PC1	i5 4690 (3.5 GHz)	16 GB	Win 10 Pro x64	7.3.5 x64	3 641 MB
PC2	i3 530 (2.93 GHz)	4 GB	Win 10 Home x64	7.3.5 x64	3 641 MB
NTB	i5 3210M (2.5 GHz)	8 GB	Win 10 Home x64	7.3.5 x64	3 641 MB
PC-J19	AMD FX-6300 (3.5 GHz)	16 GB	Win 7 Ent x64	7.3.5 x64	3 641 MB

For each method, 100 tests were conducted, which represented one testing iteration. Four testing iterations were conducted on each HW setting in order to find out whether there are any internal effects influencing acquired results. Furthermore, the existence of the "-server" parameter in the Java Virtual Machine (JVM) had to be taken into consideration. The -server parameter causes aggressive code optimization and is a standard setting in JVM benchmarks. Thus, the influence of application of -server parameter in the source code had to be verified because just-in-time compilation adds very large variance to execution time [21]. Thus, tests were conducted on single HWs also with the–server parameter applied. Based on recommendations focused on testing in the Java environment published by Georges et al. [21], the first 100 data points associated with the initialization process (“warm-up” period) were removed from the analysis. Altogether, 12800 data points were collected and consequently analyzed (these data are available in the Supplemental file S1 Dataset). The length of one test was set to 2592000 seconds of modelled time during which 432000 decision-making moments were performed. This period of 30 days (1 month of the model runtime) was selected because it provides sufficient volume of data for subsequent analysis. In average, there is a decision made every 6 second of virtual (model) time. Analyzed real-time runtimes were monitored in seconds.

### Evaluated multi-criteria decision-making methods

The performed literature review reveals that there is a lack of research papers or studies covering area of computational effectiveness of MCDM methods in general. Relevant studies mostly compare selected MCDM methods based on their application in various specialized domains, such as housing affordability [22], decision support in financial services [23], maintenance delivery in engineering industry [24], bio-energy systems [25], fuzzy logic [26], development of food products [27], or students`career preference models [28]. When the methods are mutually compared, the computational effectiveness is not included as a criterion. For instance, Thor et al. [29] focus on consistency of methods as the main criterion. Peniwati [30] offers quite complex comparison based on 16 criteria used for evaluation of 16 group decision-making methods (outranking, goal programming, MAUT, or AHP included). These range from technical to psychophysical or social ones. However, computational effectiveness is not included. There are also relevant papers published in the economic modeling domain, which might be referenced. For example a study presented by Tan, Lee and Goh [31] that focuses on empirical evaluation of MCDM techniques in business-to-business (B2B) collaboration in supply chains, or Rezaei’s paper dealing with MCDM application in reverse logistics [32] are worth noticing. The evaluation of computational effectiveness is regrettably omitted. Furthermore, it seems that there is a strong tendency to apply MCDM in a form of hybrid solutions (e.g. [33, 34], [35], [36], etc.), which makes mutual comparison of MCDM methods even more difficult, if not straightforwardly impossible. Other papers are focused on comparison of application areas, e.g. [37], but this approach leads even more towards listing of case studies for applications rather than their explicit comparison.

Lack of literature and resources on computational effectiveness might be partially related to scarce applications of MCDM in large scale simulations and models where the effectiveness and computational requirements are very important. In smaller models, other aspects of decision making such as precision and quality of decision making, robustness of the DM method, or problems related to description of task environment, usually play more important roles.

From a large number of possibilities, four multi-criteria decision-making (MCDM) methods were implemented and tested, namely the following:

- Weighted Product Method (WPM);
- Technique of Order Preference Similarity to the Ideal Solution (TOPSIS);
- Vise Kriterijumska Optimizacja I Kompromisno Resenje (VIKOR);
- Preference Ranking Organization Method for Enriched Evaluation (PROMETHEE).

These methods were selected based on their suitability for use in the model. Although decision-making support is mostly aimed at individuals or groups of individuals as the ultimate target users, it is generally perceived as desirable to minimize the participation of human element in the decision-making process and respective data preparation due to human-related issues such as subjectivity, emotions, or biases [38]. Methods requiring interaction with the user/operator or such that would be difficult to process in the model, e.g., containing pairwise comparison, evaluation scales, or the autonomous construction of fitness functions, were omitted from the testing. Examples of omitted methods are AHP, ANP, MAUT/UTA, MACBETH, ELECTRE, CBA, COMET, AIRM, GRA, NATA, PAPRIKA and others. The WSM method, as described in the following section, was omitted because of difficulties related to the handling of different units of measure and problems with multidimensional decision-making situations.

#### Weighted Sum Model and Weighted Product Model

Because there is a generally prevalent approach to start with the simplest alternative, the Weighted Sum Method (WSM) was considered as the first option for the application. WSM is a straightforward decision-making method, usually applied to one-dimensional problems. Under the assumption of *m* alternatives and *n* criteria, the best option is selected by application of the following expression (according to Fishburn [39]):

$$\begin{array}{l} A_{wsm}^{*}=max\;\sum_{i}^{j}a_{ij}w_{j}\\ i=1,\;2,\ldots ,m, \end{array}$$ (1)

A*_wsm_ denotes best alternative score,

*a*_*ij*_ denotes value of i-th alternative with consideration of j-th criterion,

*w*_*j*_ denotes weight of j-th criterion.

However, Triantaphyllou [40] states that *“*…*in single-dimensional cases*, *where all units are the same*, *WSM can be used without difficulty*. *Difficulty with this method emerges when it is applied to multi-dimensional MCDM problems*. *Then*, *in combining different dimensions*, *and consequently different units*, *the additive utility assumption is violated*.*”* For this reason, the Weighted Product Model (WPM) was used instead. The Weighted Product Model (WPM) is similar to WSM but uses multiplication instead of addition (Bridgman [41] and Miller and Starr [42]). We may suppose that a given problem is defined on *m* alternatives and *n* decision criteria. Moreover, we may assume all the criteria to be considered as benefit criteria (i.e. the higher the values, the better). Then, if a decision-maker wants to compare the two alternatives *A*_*K*_ and *A*_*L*_ (where *m* ≥ *K*, *L* ≥ 1), then the following equation needs to be calculated:

$$R\left(\frac{A_{K}}{A_{L}}\right)=\prod_{j=1}^{n}{\left(\frac{a_{Kj}}{a_{Lj}}\right)}^{w_{j}}$$ (2)

*n* is the best alternative score,

*a*_*ij*_ is actual value of i-th alternative in terms of j-th criterion,

*w*_*j*_ is weight of importance of the j-th criterion.

If the term $R\left(\frac{A_{K}}{A_{L}}\right)$ is greater or equal to one, then alternative *A*_*K*_ is more desirable than *A*_*L*_. The best alternative is better than or at least equal to all other alternatives [40]. The structure of the method eliminates any units of measure, allowing its use in both single- and multi-dimensional problems.

#### Technique for Order Preference by Similarity to Ideal Solution (TOPSIS)

The Technique for Order Preference by Similarity to Ideal Solution (TOPSIS) method was developed by Hwang and Yoon in 1981 [43]. This method is based on the concept that the chosen alternative should have the shortest Euclidean distance from the ideal solution and the farthest Euclidean distance from the negative ideal solution. The ideal solution is a hypothetical solution for which all attribute values correspond to the maximum attribute values in the database comprising the satisfying solutions; the negative ideal solution is the hypothetical solution for which all attribute values correspond to the minimum attribute values in the database. TOPSIS thus gives a solution that is not only the closest to the hypothetically best but is also the farthest from the hypothetically worst [44]. The TOPSIS procedure consists of the following steps (all adopted from [44]):

1. Calculate the normalized decision matrix from the evaluation matrix consisting of *m* alternatives and *n* criteria, with the intersection of each alternative and criteria given as f_ij_, we therefore have a matrix (f_ij_). The normalized value *r*_*ij*_ is calculated as
  $$\begin{array}{l} r_{ij}=\frac{f_{ij}}{\sqrt{\sum_{j=1}^{J}f_{ij}^{2}}}\\ j=1,\ldots ,J;\;i=1,\ldots ,n. \end{array}$$ (3)
2. Calculate the weighted normalized decision matrix. The weighted normalized value *v*_*ij*_ is calculated as
  $$\begin{array}{l} v_{ij}=w_{i}r_{ij},\\ j=1,\ldots ,J;\;i=1,\ldots ,n, \end{array}$$ (4)
  where *w*_*i*_ is the weight of the i-th attribute or criterion, and $\sum_{i=1}^{n}w_{i}=1$.
3. Determine the ideal and negative-ideal solutions
  $$A^{*}=\{v_{i}^{*},\ldots ,v_{n}^{*}\}=\left\{(\max_{j}v_{ij}|i\in I^{\prime }),(\min_{j}v_{ij}|i\in I^{\prime \prime })\right\}$$ (5)
  where *I’* is associated with benefit criteria, and *I”* is associated with cost criteria.
4. Calculate the separation measures using the *n*-dimensional Euclidean distance. The separation of each alternative from the ideal solution is given as
  $$\begin{array}{l} D_{j}^{*}=\sqrt{\sum_{i=1}^{n}{\left(v_{ij}-v_{i}^{*}\right)}^{2}}\\ j=1,\ldots ,J. \end{array}$$ (6)
  Similarly, the separation from the negative ideal solution is given as
  $$\begin{array}{l} D_{j}^{-}=\sqrt{\sum_{i=1}^{n}{\left(v_{ij}-v_{i}^{-}\right)}^{2}}\\ j=1,\ldots ,J. \end{array}$$ (7)
5. Calculate the relative closeness to the ideal solution. The relative closeness of the alternative *a*_*j*_ with respect to A^*^ is defined as
  $$\begin{array}{l} C_{j}^{*}=D_{j}^{-}/(D_{j}^{*}+D_{j}^{-})\\ j=1,\ldots ,J. \end{array}$$ (8)
6. Rank the preference order.

As is apparent from the first and second step, the TOPSIS method uses a normalization procedure, which is necessary for the comparison of different measures of units tied to single criteria. There are several usable normalization methods, e.g., distributive or ideal normalization. Weighted normalized scores are used for comparing and determining ideal and negative solutions.

#### Vise Kriterijumska Optimiyacija I Kompromisno Resenje (VIKOR)

According to Chang [45], the compromise-ranking method VIKOR is an utilizable technique for multi-criteria analysis. The method was developed for application in complex systems. The VIKOR method is grounded in ranking and selecting from the alternatives with conflicting criteria [46]. Based on the assumption that each alternative is assessed according to multiple criterion functions, the compromise ranking is conducted by comparing the measure of closeness to the ideal alternative [47], [48], [49]. There are five main steps that need to be performed in the compromise-ranking algorithm of the traditional VIKOR (procedure overtaken from [45]):

- The various alternatives are denoted as *x*^*1*^, *x*^*2*^, *…*, *x*_*m*_. For an alternative *x*_*j*_, the merit of the i-th aspect is denoted by *f*_*ij*_, i.e., *f*_*ij*_ is the value of the i-th criterion function for the alternative *x*_*j*_. Additionally, *m* is the number of alternatives, and *n* is the number of criteria.
- Determine the maximum *f*_i_^*^ and minimum *f*_*i*_^-^ values of all criterion functions, *i = 1*, *…*, *n*.

$$f_{i}^{*}=j\;\max f_{ij}=\max\left[\left(f_{ij}\right)\right|j=1,2,\ldots ,m]$$ (9)

$$f_{i}^{*-}=j\;\min f_{ij}=\min\left[\left(f_{ij}\right)\right|j=1,2,\ldots ,m]$$ (10)

- Compute the values *S*_*j*_ and *R*_*j*_, *j = 1*, *…*, *m*.

$$S_{j}=\sum_{i=1}^{n}w_{i}(f_{i}^{*}-f_{ij})/(f_{i}^{*}-f_{i}^{-})$$ (11)

$$R_{j}=i\;\max\left[w_{i}(f_{i}^{*}-f_{ij})/(f_{i}^{*}-f_{i}^{-}\right)\;|\;i=1,2,\ldots ,n]$$ (12)

*S*_*j*_ denotes utility measure for the alternative *x*_*j*_,

*R*_*j*_ denotes regret measure for the alternative *x*_*j*_,

*w*_*i*_ denotes weight of i-th criterion, which represents the relative importance of the criterion.

- Compute the values *Q*_*j*_, *j = 1*, *…*, *m*.

$$Q_{j}=\frac{v(S_{j}-S^{*})}{\left(S^{-}-S^{*}\right)}+\frac{\left(1-v)(R_{j}-R^{*}\right)}{{(R}^{-}-R^{*})}$$ (13)

$$S^{*}=j\;min\;S_{j}=\min\left[\left(S_{j}\right)\right|j=1,2,\ldots ,m]$$ (14)

$$S^{-}=j\;max\;S_{j}=\max\left[\left(S_{j}\right)\right|j=1,2,\ldots ,m]$$ (15)

$$R^{*}=j\;min\;R_{j}=\min\left[\left(R_{j}\right)\right|j=1,2,\ldots ,m]$$ (16)

$$R^{-}=j\;max\;R_{j}=\max\left[\left(R_{j}\right)\right|j=1,2,\ldots ,m]$$ (17)

where *v* is the weight for the strategy of maximum group utility, and *1-v* is the weight of the individual regret, according to Kackar [50] and Opricovic [49].

- Rank the alternatives by *Q*_*j*_. The lower the value of *Q*_*j*_ is, the better a decision is the alternative [45].

Chang [45], in the first step of the sequence, already expects normalized values of the (f_i, j_) decision matrix. Yazdani and Payam [51] and Tong et al. [52] mention the normalization of values in the first step. However, they work with vector normalization, corresponding to the distributive normalization of the TOPSIS method. This method is in contrast to Opricovic and Tzeng [49], who use linear normalization (below), which eliminates the influence of units of measure and is included in the first step of the procedure:

$$d_{ij}(f)=\frac{f_{i}^{*}-f_{ij}}{f_{i}^{*}-f_{i}^{-}}$$ (18)

#### Preference Ranking Organization Method for Enrichment Evaluation (PROMETHEE)

One of the options for evaluating a decision-making problem is the method called Preference Ranking Organization Method for Enrichment Evaluation (PROMETHEE). Currently, this method has been extended to encompass six ranking formats: PROMETHEE-I (partial ranking), PROMETHEE-II (complete ranking), PROMETHEE-III (ranking based on intervals), PROMETHEE-IV (continuous case), PROMETHEE-V (net flows and integer linear programming), and PROMETHEE-VI (representation of human brain) (see [53]).

In this experiment, PROMETHEE-II is applied; thus, the ranking of the alternatives is based on positive and negative flows. According to Ishizaka and Nemery [20], there are four possible outcomes:

1. Alternative A is better than B, if the total positive and negative flows are simultaneously better;
2. alternative A has a worse evaluation than alternative B if the total positive and negative flows are worse;
3. alternatives A and B are incomparable when one of alternatives has a better positive score but a worse negative score, and vice versa (i.e., worse positive and better negative score), and
4. two alternatives are equal in the case of matching positive and negative total flows.

PROMETHEE-II uses a ranking system based only on total net flows. This results in complete alternative evaluation, in contrast to PROMETHEE-I, which does not include a situation in which two alternatives are incomparable [20]. The methods works with the indifference and preference thresholds. While the indifference threshold for a given criterion represents the largest deviation that is considered as negligible in the comparison of two actions, the preference threshold for a given criterion corresponds to the smallest definition that a decision-maker considers as definitely important when he/she compares to actions. A thorough formal description of the first two variants of the PROMETHEE methods, namely PROMETHEE-I and PROMETHEE-II, is provided by Mateo [53]. Due to the extent of the formal description of this methods, it is left up to the reader to examine the details in the provided reference. However, implementation of the method can be found in the Supplemental file (S1 File).

## Results and Discussion

**Fig 1:**
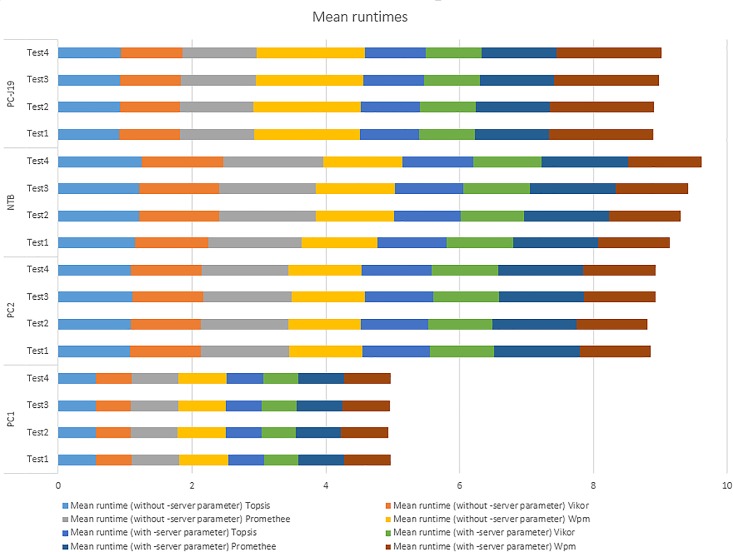
Summary of experimental results.

Table 2 presents the mean runtimes associated with particular methods and test iterations conducted on all four HWs. Although median can be generally considered as more appropriate indicator due to possible outliers contained in the dataset, these are mostly associated with the initialization process in each iteration. Since this limitation was removed based on the methodology provided by Georges et al.[21]–see section 2.2, the means are used for evaluation. Furthermore, all statistical methods applied during the verification phase are based on usage of means. A graphical representation of the acquired results is depicted in Fig 1.

**Table 2:** Mean runtimes (in sec).

		Mean runtimes (without -server parameter)				Mean runtimes (with -server parameter)			
		Topsis	Vikor	Promethee	Wpm	Topsis	Vikor	Promethee	Wpm
PC1	Test1	0.56693	0.53329	0.7101	0.7368	0.52863	0.51362	0.67828	0.6982
	Test2	0.56419	0.52506	0.69134	0.72104	0.5323	0.51814	0.67589	0.70523
	Test3	0.55898	0.52747	0.70226	0.72099	0.53576	0.52078	0.6819	0.70879
	Test4	0.56041	0.53024	0.70406	0.72725	0.53848	0.52347	0.68371	0.70927
PC2	Test1	1.07534	1.05316	1.32147	1.09817	1.00734	0.96539	1.27582	1.06349
	Test2	1.08163	1.04857	1.30821	1.09005	0.99988	0.96533	1.2504	1.06065
	Test3	1.1044	1.06458	1.31443	1.09801	1.02453	0.98377	1.26714	1.07566
	Test4	1.08128	1.05671	1.30211	1.09913	1.04008	0.99672	1.27648	1.0797
NTB	Test1	1.14361	1.09963	1.40032	1.13385	1.03314	0.99598	1.26507	1.0771
	Test2	1.20804	1.19566	1.44445	1.16943	1.00108	0.95034	1.26215	1.07288
	Test3	1.20745	1.19318	1.45507	1.17328	1.02908	0.98992	1.28114	1.08632
	Test4	1.24595	1.21836	1.49382	1.19336	1.05236	1.01607	1.30297	1.09537
PC-J19	Test1	0.9124	0.90076	1.11006	1.58886	0.88751	0.83444	1.10516	1.56151
	Test2	0.92191	0.89709	1.10144	1.6011	0.88697	0.83474	1.10244	1.56327
	Test3	0.92364	0.90514	1.12481	1.60738	0.90316	0.83406	1.12001	1.56809
	Test4	0.93713	0.91317	1.1171	1.61319	0.90797	0.83943	1.12516	1.57128

A cursory examination of the results indicates that there may be differences in terms of mean runtimes among all methods. While mean runtimes achieved by Vikor and Topsis methods seem promising, runtimes associated with Wpm and Promethee methods look inappropriate for decision-making in agent-based models. However, since some test iterations contain significant variance in runtimes, while others do not, this needs be verified. Therefore, additional statistical verification is conducted. To find out if the obtained results are influenced by certain factors, the main analyzed question is formulated as “What is the impact of Test, HW setting (only Setting hereinafter) and Method on Runtime?”. The tested hypothesis is formulated as follows “the means of runtimes are equal across groups defined by Test, Setting and Method”. Hypothesis is tested with the help of the GLM Repeated Measures model with additional post-hoc tests are applied with the help of the SPSS v23 statistical package. Firstly, the mixed model ANOVA with Test and Setting as with-in subject factors and Methods as between subject factor has been conducted. There have been four levels in each factor Test (Test1, Test2, Test3, and Test4).

**Fig 2:**
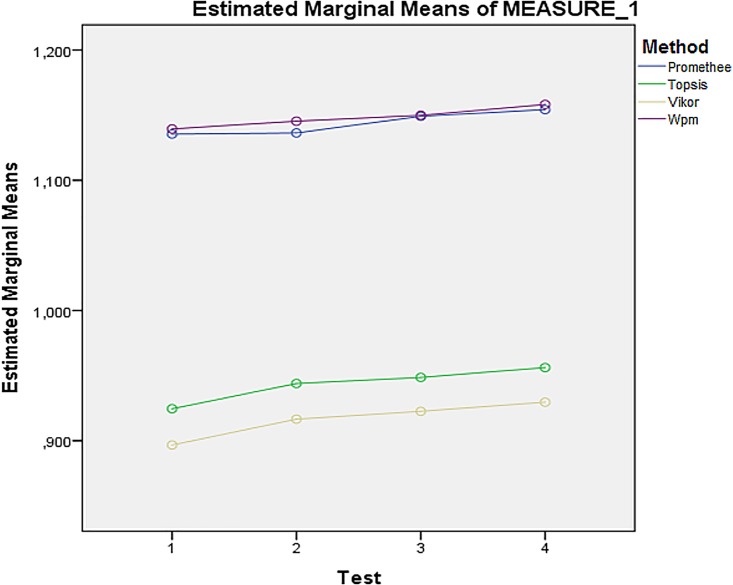
Interaction between Method and Test (without–server parameter).

The multivariate test revealed that there are significant interactions Setting * Method, and Setting * Test, Wilks' Lambda = 0.003, F (9, 394) = 1042.712, p = 0.000, and Wilks’ Lambda = 0.638, F(9, 394) = 24.512, p = 0.000 respectively. The interaction points that there were significant differences in methods across different HW settings, and in tests across different HW settings. This interaction could be explained by different hardware configuration of each computer, difference in computational effectiveness of methods and the independence of each test. The same results are acquired from Tests of Within-Subjects Effects. The interaction Setting * Test * Method is at the border line of significance. Since it is statistically difficult to interpret threefold type of interaction, it will be excluded from the analysis. The only insignificant interaction Test * Method, Wilks’ Lambda = 0.977, F (9, 394) = 1.030, p = 0.413, confirms consistency of tests, because results of tests do not differ in single methods (see Table 3). The profile plot in Fig 2 indicates that the interaction is connected with the Promethee and Wpm methods. Vikor and Topsis methods seem to have significant interaction with particular tests. Therefore, further examinations are conducted.

**Table 3:** Multivariate Tests.

Effect		Value	F	Hypothesis df	Error df	Sig.	Partial Eta Squared
Setting	Pillai's Trace	.999	119912.784^a^	3.000	394.000	0.000	.999
	Wilks' Lambda	.001	119912.784^a^	3.000	394.000	0.000	.999
	Hotelling's Trace	913.042	119912.784^a^	3.000	394.000	0.000	.999
	Roy's Largest Root	913.042	119912.784^a^	3.000	394.000	0.000	.999
Setting * Method	Pillai's Trace	1.515	134.597	9.000	1188.000	.000	.505
	Wilks' Lambda	.003	1042.712	9.000	959.043	0.000	.855
	Hotelling's Trace	156.461	6826.330	9.000	1178.000	0.000	.981
	Roy's Largest Root	155.375	20509.451^b^	3.000	396.000	0.000	.994
Test	Pillai's Trace	.208	34.434^a^	3.000	394.000	.000	.208
	Wilks' Lambda	.792	34.434^a^	3.000	394.000	.000	.208
	Hotelling's Trace	.262	34.434^a^	3.000	394.000	.000	.208
	Roy's Largest Root	.262	34.434^a^	3.000	394.000	.000	.208
Test * Method	Pillai's Trace	.023	1.029	9.000	1188.000	.415	.008
	Wilks' Lambda	.977	1.030	9.000	959.043	.413	.008
	Hotelling's Trace	.024	1.032	9.000	1178.000	.412	.008
	Roy's Largest Root	.021	2.802^b^	3.000	396.000	.040	.021
Setting * Test	Pillai's Trace	.362	24.512^a^	9.000	388.000	.000	.362
	Wilks' Lambda	.638	24.512^a^	9.000	388.000	.000	.362
	Hotelling's Trace	.569	24.512^a^	9.000	388.000	.000	.362
	Roy's Largest Root	.569	24.512^a^	9.000	388.000	.000	.362
Setting * Test * Method	Pillai's Trace	.099	1.476	27.000	1170.000	.056	.033
	Wilks' Lambda	.904	1.477	27.000	1133.802	.056	.033
	Hotelling's Trace	.103	1.477	27.000	1160.000	.055	.033
	Roy's Largest Root	.051	2.210^b^	9.000	390.000	.021	.049

Design: Intercept + Platform Within Subjects Design: Setting + Test + Setting * Test

^a^. Exact statistic

^b^. The statistic is an upper bound on F that yields a lower bound on the significance level.

Test of Between-Subject Effects reveals that influence of Methods is significant and thus methods differ from each other, F(3, 400) = 3345.173; p<0.05; Partial Eta Squared = 0.962 (see Table 4). The Post Hoc tests show that Vikor is significantly faster than the other methods. Consequently, pairwise comparisons of single methods are conducted. Results are presented in Table 5. This analysis confirms previously indicated result that there is a similarity of results acquired by Promethee and Wpm methods. Other pairs of methods, when mutually compared, differ from each other.

**Table 4:** Between-Subject Effects.

Source	Type III Sum of Squares	df	Mean Square	F	Sig.	Partial Eta Squared
Intercept	6894.874	1	6894.874	918913.096	0.000	1.000
Method	75.367	3	25.122	3348.173	.000	.962
Error	2.971	396	.008			

**Table 5:** Pairwise comparisons of methods (without–server parameter).

Method		Mean Difference (I-J)	Std. Error	Sig.^b^	95% Confidence Interval for Difference^b^	
					Lower Bound	Upper Bound
Promethee	Topsis	.200*	.003	.000	.192	.209
	Vikor	.227*	.003	.000	.219	.236
	Wpm	-.004	.003	.894	-.013	.004
Topsis	Promethee	-.200*	.003	.000	-.209	-.192
	Vikor	.027*	.003	.000	.019	.035
	Wpm	-.205*	.003	.000	-.213	-.197
Vikor	Promethee	-.227*	.003	.000	-.236	-.219
	Topsis	-.027*	.003	.000	-.035	-.019
	Wpm	-.232*	.003	.000	-.240	-.224
Wpm	Promethee	.004	.003	.894	-.004	.013
	Topsis	.205*	.003	.000	.197	.213
	Vikor	.232*	.003	.000	.224	.240

Based on estimated marginal means

*. The mean difference is significant at the .05 level.

^b^. Adjustment for multiple comparisons: Bonferroni.

Together with the aforementioned analysis several post-tests are conducted. Since Levene’s test does not confirm the null hypothesis that the error variance of the dependent variable is equal across groups, the Tukey HSD test which is commonly used cannot be applied. Therefore, the Games Howell test is used instead. This test confirms results acquired from the previous analysis (see Table 6).

**Table 6:** Games-Howell post-hoc test (without–server parameter).

		Mean Difference (I-J)	Std. Error	Sig.	95% Confidence Interval	
					Lower Bound	Upper Bound
Promethee	Topsis	.20049*	.003262	.000	.19203	.20894
	Vikor	.22744*	.003250	.000	.21901	.23586
	Wpm	-.00443	.002274	.213	-.01033	.00147
Topsis	Promethee	-.20049*	.003262	.000	-.20894	-.19203
	Vikor	.02695*	.003686	.000	.01740	.03650
	Wpm	-.20491*	.002862	.000	-.21236	-.19747
Vikor	Promethee	-.22744*	.003250	.000	-.23586	-.21901
	Topsis	-.02695*	.003686	.000	-.03650	-.01740
	Wpm	-.23186*	.002849	.000	-.23927	-.22445
Wpm	Promethee	.00443	.002274	.213	-.00147	.01033
	Topsis	.20491*	.002862	.000	.19747	.21236
	Vikor	.23186*	.002849	.000	.22445	.23927

*. The mean difference is significant at the .05 level.

**Fig 3:**
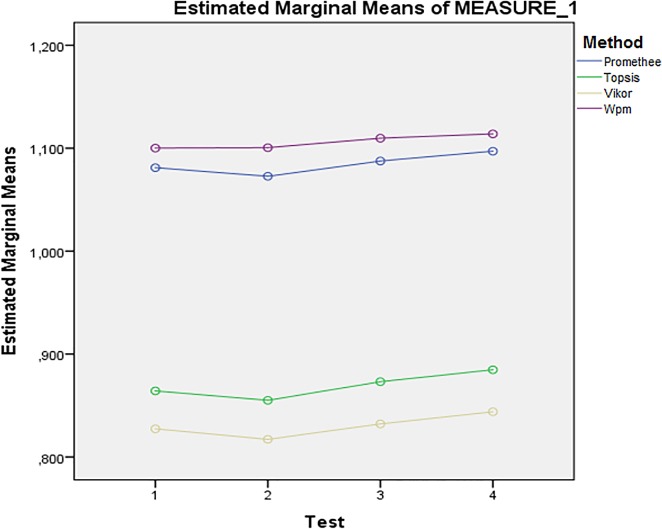
Interaction between Method and Test (with–server parameter).

As already stated, the same analysis is performed with dataset obtained during experiments with the -server parameter applied. Three out of four indicators (namely Pillai’s Trace, Wilks’ Lambda and Hotelling’s Trace) indicate insignificant interaction between Test and Method in Multivariate tests again. This is also confirmed by Test of Within-Subjects Effects. Test of Between-Subject Effects again reveals that influence of Methods is significant when the–server parameter is used and thus methods differ from each other, F(3, 400) = 8504.825; p = 0.000; Partial Eta Squared = 0.985. In opposite to previous analysis, the pairwise comparisons of methods reveal that all four methods mutually differ from each other. This is also confirmed by particular profile plot depicted in Fig 3. Similarly to the previous analysis, the Levene’s test indicates violation of an assumption of homogeneity of variances. Games Howell’s post-hoc test confirms difference with p<0.05 for every combination of methods.

**Fig 4:**
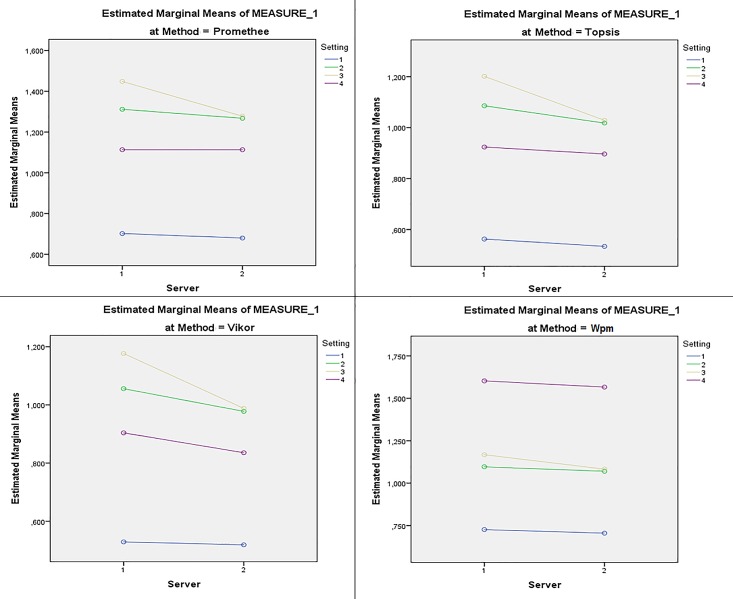
Interactions among Server * Method * Setting. (*1*): PC1; (*2*): PC2; (*3*): NTB; (*4*): PC-J19 (“1” stands for “without–server parameter”; “2” stands for “with–server parameter”).

Next part of the statistical analysis is focused on influence of the–server parameter on acquired results. Pairwise comparisons of all methods on both–server parameter states is conducted. Results are presented in Table 7. Profile plots confirm that the Vikor method represents significantly faster method for decision-making agents in agent-based simulations across all Settings and Tests. Fig 4 reveals that the–server parameter influences mean runtimes with significant effect.

**Table 7:** Server interactions.

Platform			Mean Difference (I-J)	Std. Error	Sig.^b^	95% Confidence Interval for Difference^b^	
						Lower Bound	Upper Bound
Promethee	1	2	.059*	.003	.000	.054	.064
	2	1	-.059*	.003	.000	-.064	-.054
Topsis	1	2	.074*	.003	.000	.069	.079
	2	1	-.074*	.003	.000	-.079	-.069
Vikor	1	2	.086*	.003	.000	.081	.091
	2	1	-.086*	.003	.000	-.091	-.081
Wpm	1	2	.042*	.003	.000	.037	.047
	2	1	-.042*	.003	.000	-.047	-.037

Based on estimated marginal means

“1” stands for “without–server parameter”; “2” stands for “with–server parameter”

*. The mean difference is significant at the, 05 level.

^b^. Adjustment for multiple comparisons: Least Significant Difference (equivalent to no adjustments).

**Fig 5:**
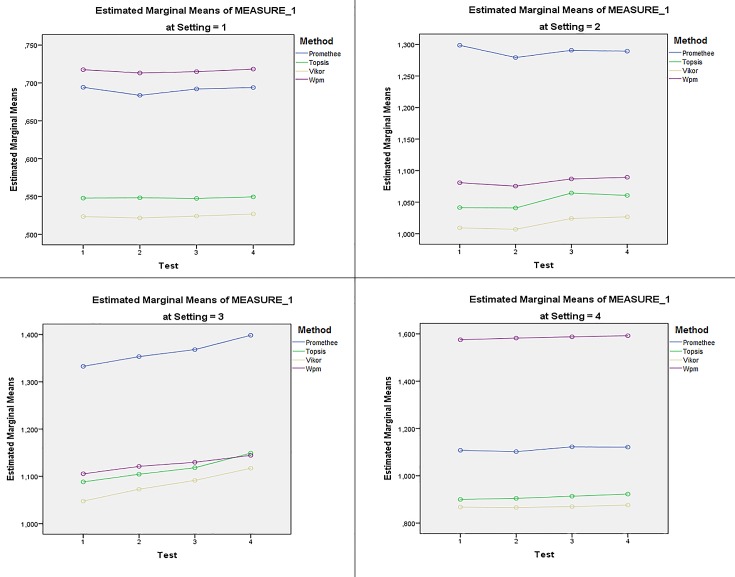
Results of interaction among Test * Method * Setting. (*1*): PC1; (*2*): PC2; (*3*): NTB; (*4*): PC-J19.

Evaluation of suitability can be summarized in Fig 5 in which all available data points were analyzed. It is apparent that the Vikor method surpasses all other methods in terms of mean runtimes at all Settings. Although the order of method suitability may change when experiments are conducted on different Settings, this variation is mostly related to Premethee and Wpm methods. The tested hypothesis thus cannot be accepted.

In order to prove that results are valid also for different sizes of population, we conducted additional experiments with 10^3, 10^4, 10^5 and 10^6 decision-making agents. This test was performed on PC with the following technical parameters: CPU: i5 4690 (3.5 GHz); RAM: 16 GB; OS: Win 10 Pro; Java: 1.8; JVM–server parameter applied; Anylogic: 7.3.4; Heap size: 512 MB (and 4GB in case of simulation with 10^6 agents–all related data are included in the Supplemental file S2 Dataset). Although the visual evaluation of line chart with experimental results indicate more or less equal growth of mean times related to all methods (no intersection is recorded), the quantitative evaluation is required. The verification is based on ANOVA test with post-hoc confirmation. Results of the ANOVA test reveal that the same order of suitability of particular methods is achieved when 10^3, 10^4, 10^5 and 10^6 decision-making agents are included in the model (F(3,396) = 229.4917, p<0.05; F(3, 396) = 13752.72, p<0.05; F(3,396) = 28883.66, p<0.05; and F(3,76) = 5774. 278, p<0.05, respectively). Sheffé’s post hoc test confirms that all means differ from each other at the alpha level 0.05 and critical value from the Fisher-Snedecor distribution in all model sizes. Hence, it is proved that achieved results are not influenced by and are valid for various agent dimensions. Results also indicate that growing number of agents results only in growing means of runtimes associated with single methods (see Table 8 with means of various model sizes). The order of methods in terms of suitability evaluated by mean runtimes remains unchanged.

**Table 8:** Mean times associated with various model sizes.

		Method		
Number of agents	Topsis	Vikor	Promethee	Wpm
10^3	0.27612	0.24031	0.34997	0.37641
10^4	2.5142	2.29611	3.36272	3.59197
10^5	25.55533	23.14144	34.48853	36.59565
10^6	256.5378	241.4143	347.8664	357.0923

Acquired results can be compared to other similar studies, which focus on comparison of various MCDM methods in different settings. For instance, in addition to studies mentioned earlier in section Calculations, Anojkumar et al. [54] provide a thorough comparative analysis of MCDM methods for industrial applications, namely FAHP-TOPSIS, FAHP-VIKOR, FAHP-ELECTRE, and FAHP-PROMETHEE. Authors conclude that “*application of VIKOR method provides valuable assistance for material selection decision-making* … *The MCDM techniques are producing significant results and also a bridge for material selection problem*”. Related research was also conducted by Mulliner et al. [55], using the WPM, WSM, AHP, TOPSIS, and COPRAS methods for comparable housing affordability decision-making problems. Mulliner and his team conclude that “*the ‘best’ (and second best) alternative obtained by all examined methods was equal but the overall ranking of all alternatives varied between methods*.” Because the comparative analysis also demonstrates that”*none of the MCDM methods are to be considered perfect*”, authors recommend the application of more than one method wherever possible.

While complex comparative analyses are quite scarce, there are however many applications using various combinations of MCDM methods, such as [56], [57], [58], [35] and [44]. It seems from a review of the literature that the actual trend in the borderline research lies in using hybrid solutions that combine two or more MCDM methods. For such applications, the results of our testing may prove to be useful, especially if the computational requirements are carefully considered.

Despite the acquired results, all methods can be used in practice. Statistically significant differences do not have to be significant in practice. The selection of the most appropriate MCDM method from among WPM, PROMETHEE, TOPSIS and VIKOR is therefore, to a large extent, matter of preference according to the given problem. However, only more important decisions should be made using MCDM methods in the case of large-scale model applications, where a large number of participating subjects are involved (in this case agents). Although simpler methods of decision-making may be less precise, the computational load associated with more sophisticated methods could in many cases outweigh any possible gain. Therefore, the strategic level of decision-making can be considered as the most suitable for MCDM application.

### Limitations and Further Research Directions

Conducted experiment is associated with some limitations that need to be explicitly stated. First, the research question is closely tied to two assumption which validity is based on the review conducted by authors. Nevertheless, it would be valuable to conduct extensive quantitative research focused on usage of platforms (software) and simulation environments (hardware) in agent-based modelling to support creation of more relevant and representative studies. Second, according to Hobbs et al. [59] a good experiment should satisfy the following conditions: (a) compare methods that are widely used, represent divergent philosophies of decision making or claimed to represent important methodological improvements, (b) address the question of appropriateness, ease of use and validity, (c) controlled, uses large samples and is replicable, (d) compares methods across a variety of problems, and (e) problems involved are realistic. Our simulation experiment satisfies all conditions except the fourth one. Thus, various models can be used in future research to confirm acquired results or further specify their validity. Third, there is an important aspect of distributed computation (using supercomputers, computational grids, high-performance networks) that is often related to large-scale models. Diverse issues related to such an implementation of the virtual environment can have a significant impact on the effectiveness of decision-making algorithms. However, there are many related problems that need to be solved, such as the fact that some of the input data used in decision-making may be necessary to share, or sub-optimal implementation may create communication bottlenecks resulting in slower data exchange. Moreover, this research direction is quite topical with many unsolved issues [60] and thus goes beyond the research question investigated in this manuscript. Nevertheless, it represents an interesting direction for further testing and future work. Finally, although unlikely, the results might be solely tied to the specific virtual environment used in the experiment. The comparison of similar experiments in different platforms and verification of the results would be very helpful and valuable.

This study has several implications. For example, the difference between small-scale models and large-scale models from the perspective of application in practice is significantly reduced. This result contributes to the development of agent-based modelling and simulation in the same way as the increase of computational power or advances in computer graphics. Based on the outcomes of this study, prospective modelers and designers might make a more qualified and intelligible choice of decision-making mechanisms for their models.

## Conclusions

Multi-criteria decision-making is formally realized with the help of various methods or algorithms. These methods might be successfully implemented in the specific subset of economic systems, i.e., agent-based computational economics in general, and in large-scale (agent-oriented) models in particular. This study conducted several experiments on four different HWs and compared four MCDM methods, WPM, TOPSIS, VIKOR, and PROMETHEE. The working hypothesis, which stated that one of the selected methods exceeds the rest in terms of computational effectiveness, is confirmed, as the acquired data demonstrated significant differences in suitability of the selected methods. Therefore, VIKOR can be recommended as the most suitable method for implementation in practice because it achieves the best results. Nevertheless, further testing under different conditions would be useful to more powerfully validate this result.

## Supporting Information

S1 Dataset Raw data. (SAV)

S2 Dataset Model sizes data. (XLSX)

S1 File Code, readme file and auxiliary xlsx file included. (ZIP)

## References

[1] Bureš V, Tučník P. Complex Agent-based Models: Application of a Constructivism in the Economic Research. E&M Economics and Management. 2014;17(3):17 10.15240/tul/001/2014-3-012

[2] Saeed K. Jay Forrester's operational approach to economics. System Dynamics Review. 2014;30(4):233–61. 10.1002/sdr.1525

[3] Kantamneni A, Brown LE, Parker G, Weaver WW. Survey of multi-agent systems for microgrid control. Engineering Applications of Artificial Intelligence. 2015;45:192–203. 10.1016/j.engappai.2015.07.005

[4] Weinberg GM. An introduction to general systems thinking / Gerald M. Weinberg Silver anniversary ed. New York: Dorset House; 2001. xxi, 279 p. p.

[5] Yu X, Xu Z. Graph-based multi-agent decision making. International Journal of Approximate Reasoning. 2012;53(4):502–12. 10.1016/j.ijar.2011.12.002

[6] Nguyen VGN, Huynh HX, Drogoul A. Modelling Multi-Criteria Decision Making Ability of Agents in Agent-Based Rice Pest Risk Assessment Model. In: Huang R, Ghorbani AA, Pasi G, Yamaguchi T, Yen NY, Jin B, editors. Active Media Technology: 8th International Conference, AMT 2012, Macau, China, December 4–7, 2012 Proceedings. Berlin, Heidelberg: Springer Berlin Heidelberg; 2012. p. 134–44.

[7] Triantaphyllou E. Multi-criteria decision making methods. Multi-criteria Decision Making Methods: A Comparative Study: Springer; 2000 p. 5–21.

[8] Peng Y, Kou G, Wang G, Shi Y. FAMCDM: A fusion approach of MCDM methods to rank multiclass classification algorithms. Omega. 2011;39(6):677–89. 10.1016/j.omega.2011.01.009

[9] Cordasco G, De Chiara R, Mancuso A, Mazzeo D, Scarano V, Spagnuolo C. A Framework for Distributing Agent-Based Simulations. In: Alexander M, D’Ambra P, Belloum A, Bosilca G, Cannataro M, Danelutto M, et al., editors. Euro-Par 2011: Parallel Processing Workshops: CCPI, CGWS, HeteroPar, HiBB, HPCVirt, HPPC, HPSS, MDGS, ProPer, Resilience, UCHPC, VHPC, Bordeaux, France, August 29 –September 2, 2011, Revised Selected Papers, Part I. Berlin, Heidelberg: Springer Berlin Heidelberg; 2012. p. 460–70.

[10] Kiran M, Richmond P, Holcombe M, Chin LS, Worth D, Greenough C, editors. FLAME: simulating large populations of agents on parallel hardware architectures. Proceedings of the 9th International Conference on Autonomous Agents and Multiagent Systems: volume 1-Volume 1; 2010: International Foundation for Autonomous Agents and Multiagent Systems.

[11] Voss A, You J-Y, Yen E, Chen H-Y, Lin S, Turner A, et al., editors. Scalable social simulation: investigating population-scale phenomena using commodity computing. e-Science (e-Science), 2010 IEEE Sixth International Conference on; 2010: IEEE.

[12] Nikolai C, Madey G. Tools of the trade: A survey of various agent based modeling platforms. Journal of Artificial Societies and Social Simulation. 2009;12(2):2.

[13] Hmida FB, Seguy A, Dupas R. MultiAgent Systems for Production Planning and Control in Supply Chains. In: Omatu S, De Paz Santana FJ, González RS, Molina MJ, Bernardos MA, Rodríguez CJM, editors. Distributed Computing and Artificial Intelligence: 9th International Conference. Berlin, Heidelberg: Springer Berlin Heidelberg; 2012. p. 205–12.

[14] Wang S, Wan J, Zhang D, Li D, Zhang C. Towards smart factory for Industry 4.0: A self-organized multi-agent system with big data based feedback and coordination. Computer Networks. 10.1016/j.comnet.2015.12.017

[15] Kagermann H, Helbig J, Hellinger A, Wahlster W. Recommendations for Implementing the Strategic Initiative INDUSTRIE 4.0: Securing the Future of German Manufacturing Industry; Final Report of the Industrie 4.0 Working Group: Forschungsunion; 2013.

[16] Klügl F. “Engineering” Agent-Based Simulation Models? In: Müller JP, Cossentino M, editors. Agent-Oriented Software Engineering XIII: 13th International Workshop, AOSE 2012, Valencia, Spain, June 4, 2012, Revised Selected Papers. Berlin, Heidelberg: Springer Berlin Heidelberg; 2013. p. 179–96.

[17] Kumari S, Singh A, Mishra N, Garza-Reyes JA. A multi-agent architecture for outsourcing SMEs manufacturing supply chain. Robotics and Computer-Integrated Manufacturing. 2015;36:36–44. 10.1016/j.rcim.2014.12.009

[18] Premm M, Kirn S. A Multiagent Systems Perspective on Industry 4.0 Supply Networks. In: Müller PJ, Ketter W, Kaminka G, Wagner G, Bulling N, editors. Multiagent System Technologies: 13th German Conference, MATES 2015, Cottbus, Germany, September 28–30, 2015, Revised Selected Papers. Cham: Springer International Publishing; 2015. p. 101–18.

[19] Tesfatsion L. Agent-based computational economics: Growing economies from the bottom up. Artificial life. 2002;8(1):55–82. 10.1162/106454602753694765 12020421

[20] Ishizaka A, Nemery P. Multi-criteria decision analysis: methods and software: John Wiley & Sons; 2013.

[21] Georges A, Buytaert D, Eeckhout L, editors. Statistically rigorous java performance evaluation. Proceedings of the 22nd annual ACM SIGPLAN conference on Object-oriented programming systems and applications; 2007; Montreal, Quebec, Canada: ACM.

[22] Mulliner E, Malys N, Maliene V. Comparative analysis of MCDM methods for the assessment of sustainable housing affordability. Omega. 2016;59, Part B:146–56. 10.1016/j.omega.2015.05.013

[23] Zopounidis C, Galariotis E, Doumpos M, Sarri S, Andriosopoulos K. Multiple criteria decision aiding for finance: An updated bibliographic survey. European Journal of Operational Research. 2015;247(2):339–48. 10.1016/j.ejor.2015.05.032

[24] Sabaei D, Erkoyuncu J, Roy R. A Review of Multi-criteria Decision Making Methods for Enhanced Maintenance Delivery. Procedia CIRP. 2015;37:30–5. 10.1016/j.procir.2015.08.086

[25] Scott JA, Ho W, Dey PK. A review of multi-criteria decision-making methods for bioenergy systems. Energy. 2012;42(1):146–56. 10.1016/j.energy.2012.03.074

[26] Celik E, Gul M, Aydin N, Gumus AT, Guneri AF. A comprehensive review of multi criteria decision making approaches based on interval type-2 fuzzy sets. Knowledge-Based Systems. 2015;85:329–41. 10.1016/j.knosys.2015.06.004

[27] Alicia HdD, Mónica MGM, Jorge JAM. Application of Multi-Criteria Decision Methods (MCDM) for the development of functional food products in Venezuela. Procedia Food Science. 2011;1:1560–7. 10.1016/j.profoo.2011.09.231

[28] Pekkaya M. Career Preference of University Students: An Application of MCDM Methods. Procedia Economics and Finance. 2015;23:249–55. 10.1016/S2212-5671(15)00486-4

[29] Thor J, Ding S-H, Kamaruddin S. Comparison of multi criteria decision making methods from the maintenance alternative selection perspective. The International Journal of Engineering and Science. 2013;2(6):27–34.

[30] Peniwati K. Criteria for evaluating group decision-making methods. Mathematical and Computer Modelling. 2007;46(7–8):935–47. 10.1016/j.mcm.2007.03.005

[31] Tan PS, Lee SSG, Goh AES. Multi-criteria decision techniques for context-aware B2B collaboration in supply chains. Decision Support Systems. 2012;52(4):779–89. 10.1016/j.dss.2011.11.013

[32] Rezaei J. A Systematic Review of Multi-criteria Decision-making Applications in Reverse Logistics. Transportation Research Procedia. 2015;10:766–76. 10.1016/j.trpro.2015.09.030

[33] Chiu W-Y, Tzeng G-H, Li H-L. A new hybrid MCDM model combining DANP with VIKOR to improve e-store business. Knowledge-Based Systems. 2013;37:48–61. 10.1016/j.knosys.2012.06.017

[34] Wang Y-L, Tzeng G-H. Brand marketing for creating brand value based on a MCDM model combining DEMATEL with ANP and VIKOR methods. Expert Systems with Applications. 2012;39(5):5600–15. 10.1016/j.eswa.2011.11.057

[35] Liu C-H, Tzeng G-H, Lee M-H. Improving tourism policy implementation–The use of hybrid MCDM models. Tourism Management. 2012;33(2):413–26. 10.1016/j.tourman.2011.05.002

[36] Lee W-S, Tu W-S. Combined MCDM techniques for exploring company value based on Modigliani–Miller theorem. Expert Systems with Applications. 2011;38(7):8037–44. 10.1016/j.eswa.2010.12.138

[37] Behzadian M, Khanmohammadi Otaghsara S, Yazdani M, Ignatius J. A state-of the-art survey of TOPSIS applications. Expert Systems with Applications. 2012;39(17):13051–69. 10.1016/j.eswa.2012.05.056

[38] Johnson DDP, Blumstein DT, Fowler JH, Haselton MG. The evolution of error: error management, cognitive constraints, and adaptive decision-making biases. Trends in Ecology & Evolution. 2013;28(8):474–81. 10.1016/j.tree.2013.05.014 23787087

[39] Fishburn PC. Letter to the editor—additive utilities with incomplete product sets: application to priorities and assignments. Operations Research. 1967;15(3):537–42.

[40] Triantaphyllou E. Multi-criteria decision making methods: a comparative study Dordrecht; Boston, Mass.: Kluwer Academic Publishers; 2000. xxviii, 288 p. p.

[41] Bridgman PW. Dimensional analysis: Yale University Press; 1922.

[42] Miller D, Starr M. Executive decisions and operations research Englewood Cliffs, 1969 Prentice-Hall, Inc: NJ.

[43] Ching-Lai H, Yoon K. Multiple attribute decision making: Methods and applications: Springer-Verlag; 1981.

[44] Rao RV. Applications of Improved MADM Methods to the Decision Making Problems of Manufacturing Environment Decision Making in Manufacturing Environment Using Graph Theory and Fuzzy Multiple Attribute Decision Making Methods. Springer Series in Advanced Manufacturing: Springer London; 2013 p. 41–135.

[45] Chang C-L. A modified VIKOR method for multiple criteria analysis. Environ Monit Assess. 2010;168(1–4):339–44. 10.1007/s10661-009-1117-0 19672684

[46] Tzeng G-H, Lin C-W, Opricovic S. Multi-criteria analysis of alternative-fuel buses for public transportation. Energy Policy. 2005;33(11):1373–83.

[47] Opricovic S. Multi-criteria Optimization of Civil Engineering Systems, Faculty of Civil Engineering, Belgrade. Table II The performance matrix. 1998.

[48] Tzeng G-H, Teng M-H, Chen J-J, Opricovic S. Multicriteria selection for a restaurant location in Taipei. International Journal of Hospitality Management. 2002;21(2):171–87.

[49] Opricovic S, Tzeng G-H. Compromise solution by MCDM methods: A comparative analysis of VIKOR and TOPSIS. European Journal of Operational Research. 2004;156(2):445–55.

[50] Kackar R. Off-line Quality-Control, Parameter Design, and the Taguchi Method-Response. Journal of Quality Technology. 1985;17(4):207–9.

[51] Yazdani M, Payam AF. A comparative study on material selection of microelectromechanical systems electrostatic actuators using Ashby, VIKOR and TOPSIS. Materials & Design. 2015;65(0):328–34. 10.1016/j.matdes.2014.09.004

[52] Tong L-I, Chen C-C, Wang C-H. Optimization of multi-response processes using the VIKOR method. Int J Adv Manuf Technol. 2007;31(11–12):1049–57. 10.1007/s00170-005-0284-6

[53] Mateo J. PROMETHEE. Multi Criteria Analysis in the Renewable Energy Industry. Green Energy and Technology: Springer London; 2012 p. 23–32.

[54] Anojkumar L, Ilangkumaran M, Sasirekha V. Comparative analysis of MCDM methods for pipe material selection in sugar industry. Expert Systems with Applications. 2014;41(6):2964–80. 10.1016/j.eswa.2013.10.028

[55] Mulliner E, Malys N, Maliene V. Comparative analysis of MCDM methods for the assessment of sustainable housing affordability. Omega. 10.1016/j.omega.2015.05.013

[56] Sánchez-Lozano JM, Teruel-Solano J, Soto-Elvira PL, Socorro García-Cascales M. Geographical Information Systems (GIS) and Multi-Criteria Decision Making (MCDM) methods for the evaluation of solar farms locations: Case study in south-eastern Spain. Renewable and Sustainable Energy Reviews. 2013;24:544–56. 10.1016/j.rser.2013.03.019

[57] Fierek S, Zak J. Planning of an Integrated Urban Transportation System based on Macro–Simulation and MCDM/A Methods. Procedia—Social and Behavioral Sciences. 2012;54:567–79. 10.1016/j.sbspro.2012.09.774

[58] Li M, Jin L, Wang J. A new MCDM method combining QFD with TOPSIS for knowledge management system selection from the user's perspective in intuitionistic fuzzy environment. Applied Soft Computing. 2014;21:28–37. 10.1016/j.asoc.2014.03.008

[59] Hobbs BF, Chankong V, Hamadeh W. An Experiment in Water Resources Planning. Water Resources Research. 1992;28(7):1767–79.

[60] Collier N, North M. Parallel agent-based simulation with Repast for High Performance Computing. Simulation. 2012:0037549712462620.

